# Cell-Based Microfluidic Device Utilizing Cell Sheet Technology

**DOI:** 10.34133/2022/9758187

**Published:** 2022-01-27

**Authors:** Katsuhisa Sakaguchi, Kei Akimoto, Masanori Takaira, Ryu-ichiro Tanaka, Tatsuya Shimizu, Shinjiro Umezu

**Affiliations:** ^1^Department of Integrative Bioscience and Biomedical Engineering, Graduate School of Advanced Science and Engineering, TWIns, Waseda University, 2-2 Wakamatsu-Cho, Shinju-Ku, Tokyo 162-8480, Japan; ^2^Department of Modern Mechanical Engineering, Graduate School of Creative Science and Engineering, Waseda University, 1-104 Totsuka-Cho, Shinju-Ku, Tokyo 169-8555, Japan; ^3^Institute of Advanced Biomedical Engineering and Science, TWIns, Tokyo Women's Medical University, 8-1 Kawada-Cho, Shinju-Ku, Tokyo 162-8666, Japan

## Abstract

The development of microelectromechanical systems has resulted in the rapid development of polydimethylpolysiloxane (PDMS) microfluidic devices for drug screening models. Various cell functions, such as the response of endothelial cells to fluids, have been elucidated using microfluidic devices. Additionally, organ-on-a-chip systems that include organs that are important for biological circulation, such as the heart, liver, pancreas, kidneys, and brain, have been developed. These organs realize the biological circulation system in a manner that cannot be reproduced by artificial organs; however, the flow channels between the organs are often artificially created by PDMS. In this study, we developed a microfluidic device consisting only of cells, by combining cell sheet technology with microtitanium wires. Microwires were placed between stacked fibroblast cell sheets, and the cell sheets adhered to each other, after which the microwires were removed leaving a luminal structure with a size approximately equal to the arteriolar size. The lumen structure was constructed using wires with diameters of 50, 100, 150, and 200 *μ*m, which were approximations of the arteriole diameters. Furthermore, using a perfusion device, we successfully perfused the luminal structure created inside the cell sheets. The results revealed that a culture solution can be supplied to a cell sheet with a very high cell density. The biofabrication technology proposed in this study can contribute to the development of organ-on-a-chip systems.

## 1. Introduction

Microfluidic devices, such as organ-on-a-chip systems, are rapidly being developed to identify drug effects and obtain biological knowledge in vitro, without conducting animal experiments [1, 2]. To date, the most commercially successful application of microfluidic devices is the inkjet printer, which is currently used in 3D bioprinters [3, 4]. Microfluidic technology has revolutionized molecular biology operations such as enzyme analysis, DNA analysis, and proteome analysis [5, 6]. In recent years, there have been many reports on the application of microchannel devices for drug screening models, and various disease models have been investigates using this device [7, 8]. Many of the microfluidic devices used as drug screening models are made of PDMS, with the channel elasticity and permeability of the culture solution differing from those in vivo. By using the drug discovery model in organ-on-chip systems, an environment closer to the living body can be created by passing the drug through blood vessels constructed by cells before reaching the target organ. Furthermore, it improves vascular function such as fibroblasts and endothelial cells lined with pericyte and secrete cytokines and antithrombotic proteins to maintain target organ function [9, 10]. Therefore, cell-based microchannels have the potential to imitate the body and improve and maintain its function. Therefore, this study investigated whether a microchannel device consisting only of cells can be created using cell sheet technology. This is a tissue engineering technique wherein cells are transformed into a sheet using a culture dish, onto which a polymer whose affinity for water changes in response to temperature is grafted. Cell sheets made from temperature-responsive culture dishes [11, 12] consist only of cells, which allow the fabrication of flow channel devices that more closely resemble living tissue. A wide variety of cell types can be used to transform cells into sheets and mimic various types of tissues [13–15]. Therefore, it is possible to construct a novel microfluidic device by combining highly functional cells, such as those of the cranial nerves, liver, and heart [16–18]. This paper proposes the development of a microchannel device using fibroblasts, which are the most basic foundation cells. Microtitanium wires were sandwiched between normal human dermal fibroblast (NHDF) cell sheets that were created using a temperature-responsive culture dish (UpCell). The cell sheets were adhered to each other, before the wires were pulled out to create the microchannels. The method of using an alginate gel tube [19], produced by coating alginate gel on gelatin fiber [20], which is produced using the coacervation method, can be applied for the production of channels with arbitrary shapes in the cell sheet. Although this method has the advantage that the shape can be easily changed, it also has the drawback that the gel is fragile and difficult to operate. Therefore, this study focused on thin metal wires, which have a more uniform shape, and are easier to handle than gels. Herein, we propose the use of titanium, which has high biocompatibility, to fabricate a microfluidic device consisting only of cells, to closely resemble the living tissue of the human body.

## 2. Materials and Methods

### 2.1. Preparation of Fibroblast Cell Sheets

NHDFs (Lonza, CC-2511) were seeded into 1.5 × 10^6^ cells in a 35 mm diameter temperature-responsive culture dishes (CellSeed, CS3007). The cells were cultured in a cell sheet medium containing 10% fetal bovine serum (Glico, 10270) and 1% penicillin/streptomycin solution (Fujifilm Wako Pure Chemical, 168023191), in a modified Dulbecco's Eagle's medium (Fujifilm Wako Pure Chemical, 043-30085). After three days of cultivation at 37°C, the cells were placed in a 20°C incubator for 20 min to detach the cells. The cell sheets were then harvested from the temperature-responsive culture dishes, as described in the literature [9, 10].

### 2.2. Fabrication of the Luminal Structures in Layered Cell Sheets Using Wires

**Figure 1:**
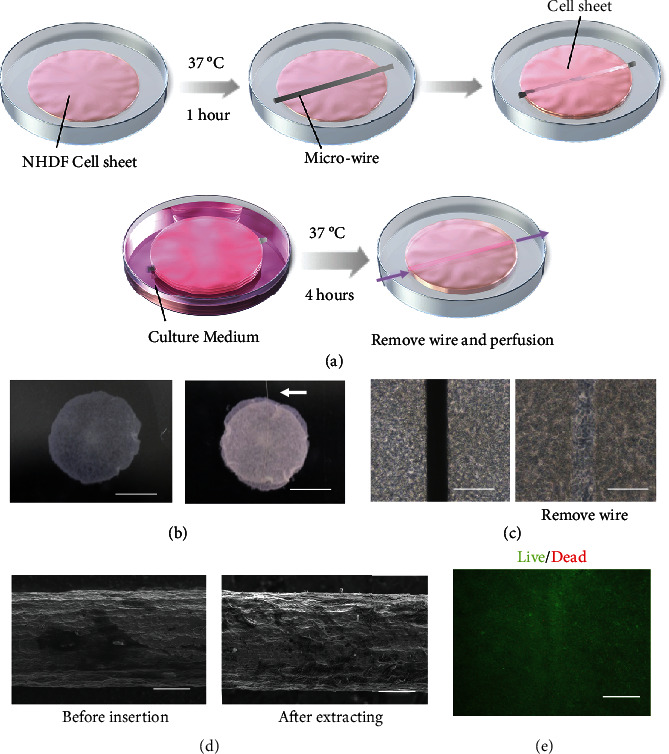
Creation of lumen structures in layered cell sheets. (a) Illustration of the process of creating the lumen structure in layered cell sheets. (b) Top view of the layered cell sheets and the layered cell sheets with the wire. The white arrow denotes the titanium wire (scale bars, 5 mm). (c) Phase contrast microscopy images before and after the wire were removed from the stacked cell sheets (scale bars, 100 *μ*m). (d) Electron microscopy images before insertion and after the extracting of the wire from the laminated cell sheet (scale bars, 20 *μ*m). (e) Performance of Live/Dead staining after removing the titanium wire (scale bars, 100 *μ*m).

The method of fabricating the luminal structures within the stacked cell sheets is shown in Figure 1. First, the harvested cell sheets were incubated for an hour at 37°C on a normal culture dish (Corning, 353001) to allow the sheets to adhere to the culture dish surface (Figure 1(a)). A titanium wire (Nilaco, TI-451166) was then placed on top of the cell sheet and a second cell sheet placed on top of the wire. Subsequently, the sheets were incubated for an hour at 37°C, followed by the placement of an additional cell sheet using the same method, to strengthen the tissue. To create strong adhesion between the sheets, the laminated cell sheets containing the wire were incubated for one day at 37°C. Pulling out the titanium wire extremely diagonally can break the cell sheet; so, it must be pulled out in parallel with the luminal structure as much as possible. The titanium wires were then extracted from the fabricated tissue. By varying the placement and diameter of the titanium wire, luminal structures with various shapes were fabricated.

### 2.3. Histological Analyses

After fabricating the luminal structure in the laminated cell sheets, the constructed tissues were fixed with 4% paraformaldehyde (Muto Pure Chemical, 3311-1). Optical coherence tomography (OCT; Santec, IVS-2000) was then used to observe the structures. Moreover, the fixed tissue was processed into 5 *μ*m-thick paraffin-embedded sections, which were then stained with hematoxylin and eosin (HE) using conventional methods, and then observed under a microscope (Nikon, Eclipse E800). Additionally, a Live/Dead staining kit (PromoCell, PK-CA707-30002) was used to determine whether the cells were alive or dead.

### 2.4. Adherence of the Vascular Endothelial Cells to the Luminal Structure

The green, fluorescent, protein-expressing human umbilical vein endothelial cells (GFP-HUVECs, Angio-Proteomie, and CAP-0001GFP) were seeded onto a HydroCell dish (CellSheed, CS2005) that inhibited cell adhesion. The cells were cultivated with titanium wire for one day at 37°C to ensure adherence of the GFP-HUVECs to the titanium wire surface. The EGM2 culture medium (Lonza, CC-3162) was changed after one day of cultivation, and the cells cultivated for two more days. The cell sheets harvested at 20°C were placed on a culture dish and left to stand for one hour. Next, GFP-HUVEC-adhered titanium wires were placed on the dish. After the second and third cell sheets were stacked, they were incubated for 1 h at 37°C. The titanium wires were then extracted after an additional day of cultivation.

### 2.5. Perfusion into the Luminal Structure

The luminal structures of the cell sheets were perfused to enable perfusion cultivation. The perfusion device was manufactured using a 3D printer (Stratasys objet260 Connex3). To prevent damage to the cell sheets during cell collection or during titanium wire extraction, the stacked cell sheets were placed on fibrin gels that were made by mixing equal amounts of 40 mg/mL fibrinogen (Sigma-Aldrich F8630-5G) in PBS and 2 U/mL thrombin (Sigma-Aldrich T4648-1KU). The titanium wire was inserted into a nozzle (Nordson, 7018433) with inner and outer diameters of 150 and 310 *μ*m, respectively. The nozzle was sandwiched between the stacked cell sheets and the titanium wire. After cultivation for one day, the microwires were extracted and the nozzle connected to a tubing pump (ISMATEC, IPC8). Black ink and microbeads (Invitrogen, F13080) were then perfused into the luminal structure at a perfusion rate of 25 *μ*L/min to confirm the flow. In addition, a pressure transducer (Edwards, TruWave) was located near the nozzle to measure the pressure applied to the constructed lumen.

## 3. Results

### 3.1. Fabrication of the Lumen Structure between Cell Sheets

A titanium microwire was sandwiched between cell sheets (Figure 1(a)). Figures 1(b) and 1(c) show the cell sheets before and after extraction of the 50 *μ*m diameter titanium wire. These images confirm that a luminal structure was created where the titanium wire was pinched. Moreover, the observation of the titanium wire after removal using electron microscopy revealed that cell adhesion did not occur on the surface of the titanium wires; thus, the titanium wire could be removed without damaging the cell sheets (Figure 1(d)). In addition, Live/Dead staining was performed to examine cell death due to wire withdrawal. The results showed that there was almost no cell death around after the wire was pulled out. (Figure 1(e)).

### 3.2. Fabrication of Luminal Structures with Different Sizes

**Figure 2:**
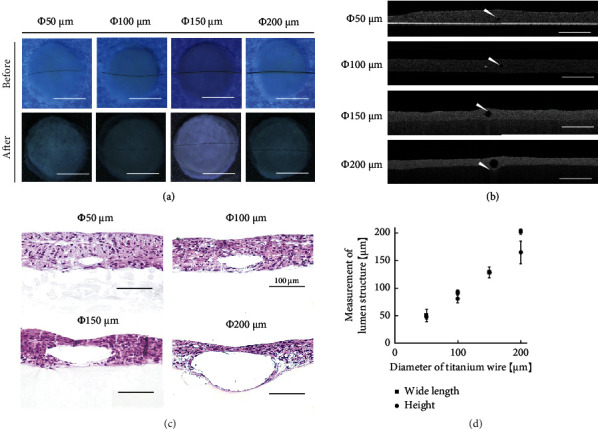
Construction of luminal structures with different diameters in the cell sheets. (a) Top view of the stacked cell sheets before and after removing the titanium wires with diameters of 50, 100, 150, and 200 *μ*m (scale bars, 5 mm). (b) Cross-sectional images captured using OCT. The white arrows indicate the lumen structures (scale bars, 500 *μ*m). (c) Histological images of the stacked cell sheets with lumen structures (scale bars, 100 *μ*m). (d) Graph showing the results obtained by the image analysis and measurement of the luminal structure size (*n* = 3). The error bars refer to ± SD.

The sandwiching of the titanium wires indicated that lumen structures were produced when the titanium wires had diameters of 50, 100, 150, and 200 *μ*m (Figure 2). Figure 2(a) shows the top view of the stacked cell sheets with and without the titanium wire. In the case of the 150 and 200 *μ*m lumen structures, it was possible to clearly observe the lumen structure that pulled the titanium wire when the titanium wires were removed. The images were obtained using OCT (Figure 2(b)) and HE staining of the cross-sections (Figure 2(c)) to visualize the luminal structures with different sizes forming in the stacked cell sheets. By quantitatively analyzing the OCT measurements, it was observed that the vertical width depended on the wire diameter. However, for the 200 *μ*m diameter sample, the height was distorted by gravity (Figure 2(d)). Thus, using this method, various blood vessel sizes were successfully created in dense cell population areas. This technology can be expanded to create blood vessel structures with diameters matching those of the target organ. However, because the luminal structure is closed after incubation for a few hours, it is necessary to culture the luminal flow and apply pressure from the luminal cavity to maintain the luminal shape.

### 3.3. Fabrication of Luminal Structures with Different Shapes

**Figure 3:**
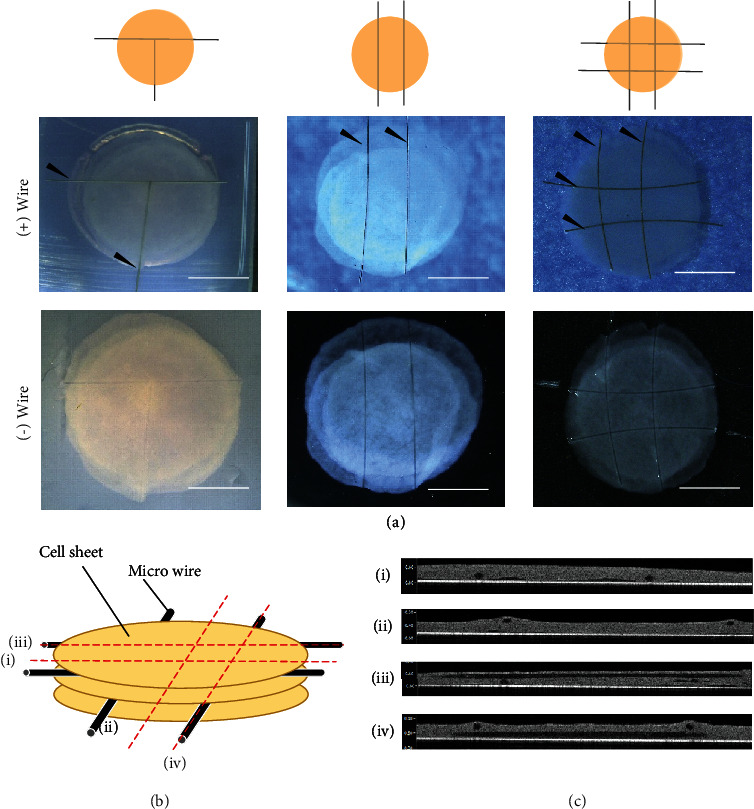
Combined cell-sheet-sandwiched titanium wires with various shapes: (a) T-shaped, T-parallel-shaped, and lattice-shaped. Each image shows an illustration with and without titanium wires. The black arrows indicate the titanium wires (scale bars, 5 mm). (b) Illustration indicates the location of cross sections. (c) Cross-sectional images captured using OCT for the lattice-shaped lumen structures. The red dotted lines indicate the observation location (i–iv).

By arranging various titanium wire formations, we succeeded in changing the shape of the wires (Figure 3). One example is the two lines or T-shape, with and without titanium wires. Additionally, by combining multiple lumen structures, we succeeded in creating a tissue with parallel and lattice-like lumen structures (Figure 3(a)). In the case of the lattice-like lumen structures, the luminal structure was investigated using OCT for more detailed observations (Figures 3(b) and 3(c)).

### 3.4. Adhesion of GFP-HUVECs to the Lumen of the Fabricated Vessels

**Figure 4:**
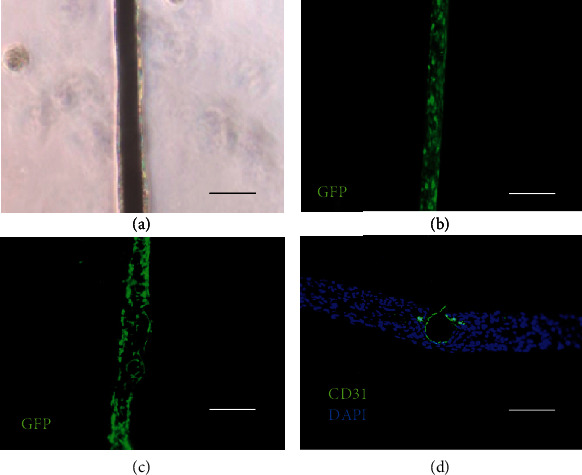
Vascular endothelialization of the luminal structures in the stacked cell sheets: (a) GFP-HUVECs adhered to titanium surface, (b) fluorescence microscopy images of the GFP-HUVEC adhered to the titanium wire surface, (c) fluorescence microscopy images of the laminated cell sheet after removing the titanium wire engrafted with the GFP-HUVEC, and (d) fluorescence microscopy images of a section of the laminated cell sheet after removing the titanium wire onto which the GFP-HUVECs were engrafted (CD31 staining (green), and DAPI staining (blue) was performed with all scale bars equal to 100 *μ*m).

The GFP-HUVECs were adhered to the surface of the titanium wires without prior coating applied to the wire surfaces, as explained in Section 2.4. Figures 4(a) and 4(b) show the GFP-HUVEC adhesion results on the titanium wires as a phase-contrast image and fluorescence image. The GFP-HUVEC-grafted titanium wire was then placed between the NHDF cell sheets and transferred to the inner lumen of the cell sheet. It was examined that the GFP-HUVECs between the cell sheets peeled off from the titanium wire (Figure 4(c)). Figure 4(d) shows the histological analysis of the cross-sections stained with anti-CD31 and DAPI (4,6-diamidino-2-phenylindole). Based on this analysis, it was concluded that the GFP-HUVECs adhered to the titanium wire were transferred to the surface of the lumen structure.

### 3.5. Perfusion into the Luminal Structures in the Tissue

**Figure 5:**
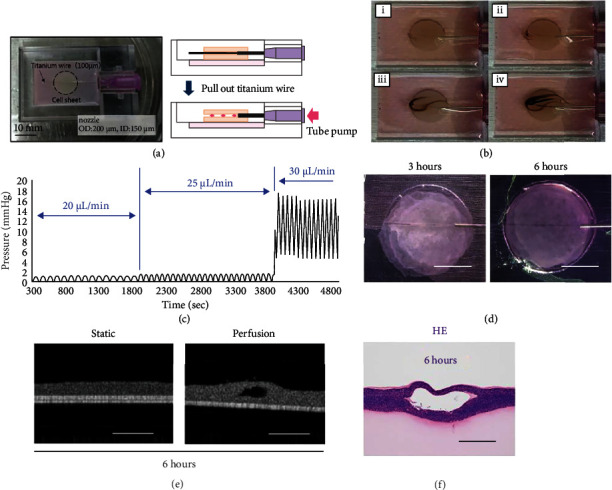
Perfusion into the luminal structure constructed in a stacked cell sheet: (a) top view photograph of the perfusion device (scale bar is 10 mm). The right image is a schematic illustration of the perfusion device's cross-section. The illustrations show the process of removing the titanium wire. (b) Perfusion of the black ink through lumen structure. The perfusion condition times shown in figures (i)–(iv) are 0, 30, 60, and 90 s, respectively. (c) Graph measuring perfusion pressure. It was measured by increasing the perfusion rate to 20, 25, and 30 *μ*L/min. (d) Photographs of the long-term perfusions at 3 h and 6 h, respectively (scale bars, 5 mm). (e) OCT images after 6 hours in static culture (left) and perfusion culture (right) (scale bar, 200 *μ*m). (f) HE-stained cross-section of the tissue with the lumen structure after 6 h perfusion (scale bar, 50 *μ*m).

Perfusion experiments were conducted to test whether perfusion was possible and to confirm that the luminal structure was continuously maintained. As shown in Figure 5, the black ink flowed into the lumen without leaking and exited through the outlet. Figure 5(a) shows the top of the perfusion culture device as well as the side view illustration. Figure 5(b) shows images of the black ink being pumped into the inner lumen structure of the laminated cell sheet (the images were captured every 5 s). Microbeads also were perfused in the structure (Supplementary video available here). In addition, the pressure was measured by gradually increasing the flow rate to 20, 25, and 30 *μ*L/min in order to investigate the durability of the lumen structure. As a result, it was confirmed that the pressure increased sharply at 30 *μ*L/min, the cell sheet was torn and covered with the nozzle, and the pressure increased further (Figure 5(c)). The culture medium was then continually perfused over an extended period to determine the durability of the luminal structure. Figure 5(d) shows the results obtained after 3 h and 6 h of perfusion. As can be seen, the shape was maintained without breaking the luminal structure. Thus, it was concluded that a high-density cell tissue with a perfusable lumen structure had been successfully produced. Figure 5(e) shows OCT images of statically cultured cell sheets (left) and perfusion-cultured cell sheets. After 6 hours of culture, it was found that the lumen was occluded by static culture, and the lumen structure could be maintained by perfusion. Observation of HE staining also showed that the luminal structure was maintained (Figure 5(f)).

## 4. Discussion

In this study, a microfluidic device consisting only of cells was developed by combining cell sheet technology, microwires, and perfusion bioreactors. The results revealed that various diameters and shapes could be created. By attaching endothelial cells, a cell structure similar to that of a living body was created. In future studies, this method can be used to construct drug screening models using a vascular network. Furthermore, if the cell sheet contains endothelial cells to provide a network, the culture solution would flow from the constructed luminal structure to the network. There is a great possibility of three-dimensional tissue construction in a short period of time. In order to realize three-dimensional tissue construction, it is first necessary to increase the durability of the luminal structure. This requires the development of a novel culture medium that produces high proliferative and high extracellular matrix production. In the future, long-term perfusion will be realized using such a culture solution.

Although it was possible to create a luminal structure coated with endothelial cells, this cell-based microfluidic devices have not exhibited the same functional and physical property values as vascular networks because of the polarity that exists in the functionality of endothelial cells. In this study, the cell-cell adhesion was stronger than the cell-titanium adhesion; therefore, the adherent surface of the GFP-HUVEC may exhibit in vivo vascular-like polarity during this process. Shamloo et al. investigated the polarity of endothelial cells in microfluidic devices [21]. By implementing their method in this study, we investigated the similarity of endothelial cells to those of the human body, and whether they could will maintain vascular functions such as antithrombogenicity. As the next evaluation, the diffusion value is an important physical property of the vascular network. The size and volume of the protein fluid leaking from the vascular network must be investigated similar to that of the living body, and a determination made on how close they are to those of normal blood vessels, or to those of the fragile vascular network present in cancerous tissue [22]. Therefore, it will be evaluated as diffusion as a future study.

Previous studies have only reported on the creation of capillaries in stacked cell sheets [23, 24]. Furthermore, the previous studies required as long as seven days for construction, the method of creating a microfluidic device consisting only of cells requires a significantly short construction duration of one day, which is a luminal structure in the layered cell sheets. Therefore, it is an innovative technology for constructing 3D tissues. In this study, the perfusion rate was 25 *μ*L/min, which is approximately equal to the blood flow rate in the human body [25]. Therefore, using this technique, perfusion at the blood flow rate is possible, which has been difficult to achieve in previous studies. In this experiment, the verification has a limited tissue size and a very short time. In future studies, the rapid development of 3D tissue construction technology should be investigated using this method to create microfluidic devices that allow long-time perfusion. Furthermore, a more advanced three-dimensional structure could be constructed by combining cell-based sensors [26].

## 5. Conclusion

In this study, a microchannel device consisting of cells only was successfully developed. Moreover, a luminal structure and blood vessel-like structure with arterioles and an arbitrary thickness of 50-200 *μ*m were developed inside the cell tissue. Furthermore, using a novel device, we successfully perfused the luminal structure created inside the cell tissue. Additionally, perfusion was performed at a flow rate that had been difficult to achieve in previous studies. We believe that this method can contribute to the development of drug screening models and new organ-on-a-chip systems.

## Data Availability

The data used to support the findings of this study are available from the corresponding author upon request.

## Supplementary Materials

Supplementary Materials Supplementary video: the video shows the green fluorescent mircobeads flowing into the inner lumen structure of the laminated cell sheet.

## References

[1] Zhang B., Korolj A., Lai B. F. L., Radisic M., Radisic M. (2018). Advances in organ-on-a-chip engineering. *Nature Reviews Materials*.

[2] Wu Q., Liu J., Wang X. (2020). Organ-on-a-chip: recent breakthroughs and future prospects. *BioMedical Engineering OnLine*.

[3] Yu F., Choudhury D. (2019). Microfluidic bioprinting for organ-on-a-chip models. *Drug Discovery Today*.

[4] Umezu S., Ohmori H. (2014). Characteristics on micro-biofabrication by patterning with electrostatically injected droplet. *CIRP Annals-Manufacturing Technology*.

[5] Lukyanenko K. A., Denisov I. A., Yakimov A. S. (2017). Analytical enzymatic reactions in microfluidic chips. *Applied Biochemistry and Microbiology*.

[6] Bruijns B., van Asten A., Tiggelaar R., Gardeniers H. (2016). Microfluidic devices for forensic DNA analysis: a review. *Biosensors*.

[7] Smoak M. M., Pearce H. A., Mikos A. G. (2019). Microfluidic devices for disease modeling in muscle tissue. *Biomaterials*.

[8] Kimura H., Sakai Y., Fujii T. (2018). Organ/body-on-a-chip based on microfluidic technology for drug discovery. *Drug Metabolism and Pharmacokinetics*.

[9] Liu C., Ge H., Liu B. (2019). Targeting pericyte–endothelial cell crosstalk by circular RNA-cPWWP2A inhibition aggravates diabetes-induced microvascular dysfunction. *Proceedings of the National Academy of Sciences of the United States of America*.

[10] Eilken M. H., Diéguez-Hurtado R., Schmidt I. (2017). Pericytes regulate VEGF-induced endothelial sprouting through VEGFR1. *Nature Communications*.

[11] Okano T., Yamada N., Sakai H., Sakurai Y. (1993). A novel recovery system for cultured cells using plasma-treated polystyrene dishes grafted with poly (N-isopropylacrylamide). *Journal of Biomedical Materials Research*.

[12] Yang J., Yamato M., Shimizu T. (2007). Reconstruction of functional tissues with cell sheet engineering. *Biomaterials*.

[13] Nishida K., Yamato M., Hayashida Y. (2004). Corneal reconstruction with tissue-engineered cell sheets composed of autologous oral mucosal epithelium. *The New England Journal of Medicine*.

[14] Ohki T., Yamato M., Murakami D. (2006). Treatment of oesophageal ulcerations using endoscopic transplantation of tissue-engineered autologous oral mucosal epithelial cell sheets in a canine model. *Gut*.

[15] Miyahara Y., Nagaya N., Kataoka M. (2006). Monolayered mesenchymal stem cells repair scarred myocardium after myocardial infarction. *Nature Medicine*.

[16] Ryu B., Sekine H., Homma J. (2020). Allogeneic adipose-derived mesenchymal stem cell sheet that produces neurological improvement with angiogenesis and neurogenesis in a rat stroke model. *Journal of Neurosurgery*.

[17] Ohashi K., Yokoyama T., Yamato M. (2007). Engineering functional two- and three-dimensional liver systems _in vivo_ using hepatic tissue sheets. *Nature Medicine*.

[18] Shimizu T., Sekine H., Yang J. (2006). Polysurgery of cell sheet grafts overcomes diffusion limits to produce thick, vascularized myocardial tissues. *The FASEB Journal*.

[19] Arai T., Tanaka R., Sakaguchi K., Umezu S. (2017). Fabrication of micro-gelatin fiber utilizing coacervation method. *Artificial Life Robotics*.

[20] Sakaguchi K., Arai T., Shimizu T., Umezu S. (2017). Fabrication of micro-alginate gel tubes utilizing micro-gelatin fibers. *Japanese Journal of Applied Physics*.

[21] Shamloo A., Ma N., Poo M., Sohn L. L., Heilshorn S. C. (2008). Endothelial cell polarization and chemotaxis in a microfluidic device. *Lab on a Chip*.

[22] Dewhirst M. W., Secomb T. W. (2017). Transport of drugs from blood vessels to tumour tissue. *Nature Reviews Cancer*.

[23] Sakaguchi K., Shimizu T., Horaguchi S. (2013). In vitro engineering of vascularized tissue surrogates. *Scientific Reports*.

[24] Sekine H., Shimizu T., Sakaguchi K. (2013). _In vitro_ fabrication of functional three-dimensional tissues with perfusable blood vessels. *Nature Communications*.

[25] Thomas B., Sumam K. S. (2016). Blood flow in human arterial system-a review. *Procedia Technology*.

[26] Oda H., Kihara K., Morimoto Y., Takeuchi S. (2021). Cell-based biohybrid sensor device for chemical source direction estimation. *Cyborg and Bionic Systems*.

